# The Immune Cell Atlas of “Longevity Molecular Tag”: Identification of Principal Immune Cell Subsets and Their Underlying Molecular Regulatory Mechanisms

**DOI:** 10.1111/acel.70431

**Published:** 2026-03-05

**Authors:** Zhiling Zhang, Huabin Su, Shihui Fu, Fansen Ji, Liuguan Liang, Wanlu Song, Caiyou Hu, Liuxiang Wei, Erping Long, Yang Lin, Xiaolin Ni

**Affiliations:** ^1^ Department of Pharmacy Beijing Anzhen Hospital, Capital Medical University Beijing China; ^2^ Jiangbin Hospital of Guangxi Zhuang Autonomous Region Nanning China; ^3^ Department of Cardiology, Hainan Geriatric Disease Clinical Medical Research Center, Hainan Branch of China Geriatric Disease Clinical Research Center Hainan Hospital of Chinese PLA General Hospital Sanya China; ^4^ Department of Geriatric Cardiology Chinese PLA General Hospital Beijing China; ^5^ Key Laboratory of Digital Intelligence Hepatology (Ministry of Education), School of Clinical Medicine, Hepatopancreatobiliary Center, Beijing Tsinghua Changgung Hospital Tsinghua University Beijing China; ^6^ Guangxi University of Chinese Medicine Nanning China; ^7^ State Key Laboratory of Respiratory Health and Multimorbidity, Institute of Basic Medical Sciences & School of Basic Medicine Chinese Academy of Medical Sciences & Peking Union Medical College Beijing China

**Keywords:** centenarian, cytotoxicity, immunosenescence, Scissor algorithm, scRNA‐seq

## Abstract

Immunosenescence represents a critical aspect of the aging process. Centenarians, serving as a nature model of “healthy aging,” demonstrate a distinctive immune “compensatory adaptation” mechanism that contributes to the maintenance of immune homeostasis. However, the specific immune cell subsets involved and the molecular mechanisms underlying these phenotypic traits remain incompletely understood. In this study, we integrated single‐cell RNA sequencing data spanning the entire lifespan of East Asian populations with bulk transcriptomic data from a centenarian cohort in Guangxi. Utilizing the Scissor algorithm, we identified immune cell subpopulations positively (Scissor^+^) and negatively (Scissor^−^) associated with longevity phenotypes, thereby constructing an immune cell atlas of “Longevity Molecular Tag.” Our findings indicate that Scissor^+^ cells predominantly comprise natural killer (NK) cells, CD8^+^ T cells, and γδ T cells, characterized by enhanced cytotoxic and immunomodulatory functions. Conversely, Scissor^−^ cells mainly include CD4^+^ T cells, B cells, and dendritic cells (DCs), which are linked to inflammatory signaling pathways and Th17/Th1 differentiation. Trajectory analysis elucidated the differentiation pathways of NK, CD8^+^ T cells, CD4^+^ T cells, and B cells. Differentially expressed genes were enriched in pathways such as NF‐κB signaling, T cell receptor signaling, and NK cell cytotoxicity. Furthermore, co‐localization analysis revealed five eQTL‐colocalized events (rs3793537–GLIPR2/CD72/TLN1 and rs8019902–TRDV2/TRDC) associated with longevity. Collectively, these results suggest that centenarians achieve immune equilibrium by remodeling cytotoxic immune lineages and finely tuning inflammatory responses, thereby promoting health span and longevity. This study offers novel insights into potential strategies for modulating immunosenescence.

## Graphical abstract

This study aims to develop an immune cell atlas of the “Longevity Molecular Tags” by employing the Scissor algorithm to identify key immune cell subsets associated with longevity phenotypes. Subsequently, the underlying immune molecular mechanisms contributing to the longevity‐associated immune cell subsets were investigated through differential gene expression analysis, pseudotime analysis, and co‐localization analysis.

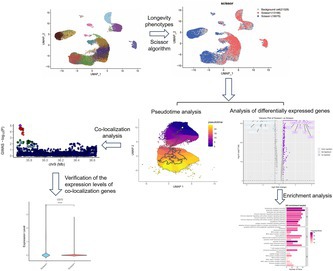

## 1 Introduction

Immunosenescence represents a fundamental characteristic of the aging process, characterized by the gradual deterioration and dysregulation of the immune system, which constitutes a significant health risk for the elderly population. This phenomenon markedly increases vulnerability to infectious diseases, diminishes vaccine efficacy, and elevates the incidence of age‐associated diseases (AADs) and malignancies (Liu et al. 2023; Lian et al. 2020). Central to the underlying pathological and physiological alterations is a state of systemic chronic low‐grade inflammation, commonly referred to as inflammaging, which serves as a pivotal driver of various AADs (Santoro et al. 2021; Barbe‐Tuana et al. 2020). At the cellular level, notable senescence‐like features emerge within the adaptive immune compartment—particularly in T and B lymphocytes—as well as in key innate immune cells such as natural killer (NK) cells. For example, diminished expression of IL7RA (IL‐7Rα) has been observed in effector‐memory and terminally differentiated CD8^+^ T cells in the context of aging. This reduction is associated with attenuated STAT5 phosphorylation following IL‐7 stimulation, inadequate upregulation of BCL2, and decreased cell survival. These findings suggest a functional immunosenescence‐associated phenotype characterized by disruption of the IL‐7Rα–IL‐7–STAT5–BCL2 signaling pathway, leading to impaired homeostatic maintenance of T cells (Kim et al. 2006; Janelle et al. 2021). A separate investigation has demonstrated an elevation in the mRNA and protein levels of tumor necrosis factor‐alpha (TNF‐α) within resting B cells derived from aged mice. Importantly, administration of anti‐TNF‐α antibodies was found to restore B cell functionality in vitro, whereas in vivo treatment with anti‐TNF‐α improved the performance of follicular B cells in aged mice (Frasca et al. 2012; Landin et al. 2011). These findings suggest a causal relationship between the increased TNF‐α mRNA/protein expression and the functional impairment observed in aged B cells. The functional capacity of senescent NK cells is also altered, characterized by diminished cytotoxicity, modified cytokine secretion profiles (e.g., IFN‐γ, tumor necrosis factor‐alpha [TNF‐α]), and disrupted receptor expression patterns (Mocchegiani and Malavolta 2004). Collectively, these dysfunctional immune cells contribute to a deleterious immune microenvironment through persistent or aberrant secretion of pro‐inflammatory mediators (e.g., interleukin‐7RA [IL‐7Rα], TNF‐α), autoantibodies, and cytotoxic factors. This milieu is implicated in the pathogenesis of insulin resistance (Shimi et al. 2024), endothelial dysfunction, vascular lipid accumulation, plaque instability, and exacerbation of neuroinflammatory processes. These mechanisms directly facilitate the onset and progression of AADs such as type 2 diabetes mellitus (Sim et al. 2023), atherosclerosis and its sequelae (including myocardial infarction and stroke), neurodegenerative disorders (notably Alzheimer's disease), and osteoarthritis (Costantini et al. 2018). Consequently, an in‐depth elucidation of immunosenescence, particularly the cellular and molecular underpinnings of inflammaging, is essential for advancing the understanding of aging pathophysiology and for the identification of potential therapeutic targets aimed at promoting healthspan.

Centenarians, regarded as paradigms of “healthy aging,” demonstrate distinctive compensatory adaptive mechanisms within their immune systems. Rather than merely postponing immunosenescence, they undergo a process of immune function remodeling and rebalancing throughout aging, particularly in the maintenance of adaptive immune homeostasis (Csaba 2019). This adaptability is evident at multiple levels, including enhanced pathogen clearance, more effective anti‐tumor immune surveillance, and improved tolerance to self‐antigens (Santoro et al. 2021). Consequently, a systematic analysis of the dynamic alterations in immune cell composition, status, and function in centenarians, compared to those in normally aging individuals, holds significant value for elucidating the boundaries of physiological aging in humans and identifying immune protective mechanisms linked to longevity (Wang, Zhang, et al. 2025). Guangxi province in China, notable for its high proportion of centenarians, predominantly comprises healthy centenarians who are native residents with limited exposure to artificial interventions such as medical treatments or pharmaceuticals, thereby serving as a natural model for longevity research (Deng et al. 2024; Huang et al. 2020). Accordingly, investigating longevity‐associated immune cell populations in centenarians from Guangxi offers a highly representative framework for such studies.

Single‐cell RNA sequencing (scRNA‐seq) technology, with its powerful ability to unbiasedly analyze cellular heterogeneity, has become a cutting‐edge tool for studying the aging immune microenvironment (Wang, Zhang, et al. 2025). Several scRNA‐seq studies on centenarians have revealed significant immune features that distinguish them from ordinary elderly individuals (Dong et al. 2022). These studies show that in the peripheral blood of centenarians, NK cells (especially the CD56^dim^ cytotoxic subpopulation) not only maintain their relative proportion but also exhibit stronger cytotoxic activity (e.g., higher expression of perforin, granzyme B‐related genes) and cytokine production capacity (e.g., IFN‐γ) (Wang, Zhang, et al. 2025). Anti‐aging‐associated receptors (e.g., NKG2D) are also more stable in expression. The T cell repertoire in centenarians shows better maintenance of diversity (Tedone et al. 2019). Among CD8^+^ T cells, subpopulations with stronger effector functions and proliferative potential (e.g., effector memory T cells [TEM], stem cell‐like memory T cells [TSCM]) are more abundant or functionally superior, whereas the accumulation of terminally differentiated, exhausted T cells (e.g., TEMRA) is less frequent (Wang, Zhang, et al. 2025). CD4^+^ T cells display stronger helper functions and a more balanced Th1/Th2/Treg (regulatory T cell) ratio, with Treg cells showing more precise suppression without excessive immune suppression (Hashimoto et al. 2019). Additionally, some investigations report that centenarians sustain greater diversity within their B cell repertoire, characterized by an increased proportion of plasma cells and plasmablasts, as well as potentially superior antibody quality, exemplified by enhanced affinity maturation (Colonna‐Romano et al. 2010).

Although scRNA‐seq can provide high‐resolution cellular maps, current scRNA‐seq investigations of centenarians are predominantly descriptive in nature and frequently fail to directly associate distinct single‐cell states with the longevity phenotype. Moreover, these studies often lack a systematic integration of dynamic and genetic data to identify key mechanistic drivers. To address these gaps, this study first integrates single‐cell data across cohorts and ethnicities (including the Shanghai Pudong cohort and Japanese supercentenarians data, *n* = 68) to construct a single‐cell aging atlas for East Asian aging populations. Then, based on the bulk RNA‐seq data of peripheral blood mononuclear cells (PBMCs) from the Guangxi centenarian cohort (34 centenarians and 16 age‐matched controls), differential expression analysis is performed to identify genes significantly associated with the longevity phenotype, constructing a “Longevity Molecular Tags.” Subsequently, the Scissor algorithm (Sun et al. 2022) is used to calculate the gene expression similarity of each cell subpopulation in the single‐cell aging atlas to this signature, unbiasedly selecting key immune cell subpopulations and their core regulatory networks that are significantly positively (Scissor^+^ cells) or negatively (Scissor^−^ cells) correlated with the longevity phenotype. Expanding upon the phenotype‐guided cell selection, we subsequently conducted pseudotime analyses to offer dynamic insights into the differentiation trajectories of subpopulations associated with longevity. Additionally, we performed colocalization analyses to prioritize candidate genetic variants that influence gene expression, thereby enhancing the mechanistic credibility of the identified longevity‐related pathways and providing a more precise framework for subsequent experimental validation.

## 2 Materials and Methods

### 2.1 Human Blood Sample Collection and Storage

This research received approval from the Ethics Committee of the Institute of Basic Medical Sciences, Chinese Academy of Medical Sciences (approval number ZS2024001). The study enrolled 34 centenarians and 16 regional‐matched controls (mean age: 52.81 ± 9.78 years), who exhibited no advanced pathological conditions, including cardiovascular diseases, neurodegenerative disorders, or cancers. These participants were selected from a larger cohort comprising 1363 individuals aged 90–110 years and 692 controls aged 40–80 years, all recruited from the Guangxi Zhuang Autonomous Region, China. Written informed consent was obtained from all participants prior to inclusion. Morning blood samples were collected from both centenarians and control subjects using tubes containing ethylenediaminetetraacetic acid (EDTA). Demographic data (gender and age) alongside clinical parameters—including basic metabolic panel, liver and renal function tests, blood glucose levels, lipid profiles, and blood pressure measurements—were assessed to confirm the absence of overt clinical indicators of disease in the sampled individuals.

### 2.2 Isolation of PBMCs and RNA Sequencing

PBMCs were isolated via density gradient centrifugation at 4000 rpm for 10 min using Ficoll‐Paque PLUS within 4 h of sample collection. Total RNA was subsequently extracted from the isolated PBMCs employing the TRIzol reagent kit (Invitrogen, Carlsbad, CA, USA) in accordance with the manufacturer's instructions. The integrity and quality of the extracted RNA were evaluated using an Agilent 2100 Bioanalyzer (Agilent Technologies, Palo Alto, CA, USA) and further verified through RNase‐free agarose gel electrophoresis. Messenger RNA (mRNA) was enriched utilizing oligo(dT) beads, followed by fragmentation into short segments using a fragmentation buffer. These fragments were then reverse transcribed into complementary DNA (cDNA) employing the NEBNext Ultra RNA Library Prep Kit for Illumina (NEB #7530; New England Biolabs, Ipswich, MA, USA). The resulting purified double‐stranded cDNA fragments underwent end repair and adenylation, after which Illumina sequencing adapters were ligated. The ligation products were purified using AMPure XP Beads at a 1.0× ratio. Subsequently, polymerase chain reaction (PCR) amplification was conducted to enrich the library. The prepared sequencing libraries derived from PBMCs were then subjected to high‐throughput sequencing on an Illumina Nova‐Seq platform by CapitalBio Technology (Beijing, China).

### 2.3 Single Cell RNA Sequencing Data Collection and Integration

Single‐cell transcriptomic data from 56 healthy individuals aged from birth to over 90 years were acquired from the Shanghai Pudong Cohort (NCT05206643) (Synapse: syn61609846) (Wang, Li, et al. 2025). Additionally, data from seven supercentenarians and five control subjects aged between their 50s and 80s were retrieved from a publicly available database (http://gerg.gsc.riken.jp/SC2018/). The raw datasets were downloaded and processed using the Read10x function within the Seurat framework (Satija et al. 2015). Standard preprocessing procedures were applied to derive the number of detected features (nFeature) and the percentage of mitochondrial RNA, which served as quality control metrics.

### 2.4 Batch Effect Correction and Cell Subtype Annotations

To integrate cells from multiple datasets into a unified space for unsupervised clustering, we applied the Harmony algorithm to correct for batch effects (Korsunsky et al. 2019). Initially, data normalization and scaling were performed using the NormalizeData and ScaleData functions within the Seurat software package to standardize and homogenize the dataset. During the scaling process, the FindVariableFeatures function was employed to identify the top 2000 highly variable genes (HVGs), which were retained for downstream analyses after excluding all ribosomal and mitochondrial genes. Subsequently, in the dimensionality reduction step of the integrated data, an elbow plot was utilized to determine the optimal number of principal components (PCs) to retain, thereby capturing the majority of data variance. The PCs corresponding to the inflection point of the elbow plot curve, as well as those demonstrating a plateauing trend, were selected for clustering in the dimensionality reduction procedure.

Subsequently, a nearest neighbor graph was constructed to facilitate cluster identification using the Louvain algorithm, with the resolution parameter set at 0.1, which yielded eight distinct cell clusters. The Seurat sub‐function FindAllMarkers was then employed to determine marker genes for each cluster, applying the following parameters: only.pos = TRUE, min.pct = 0.25, logfc.threshold = 0.25, and test.use = “wilcox.” Drawing upon prior studies (Hashimoto et al. 2019; Wang, Li, et al. 2025; Zhu et al. 2023) and data from the ACT single‐cell database (http://xteam.xbio.top/ACT/), the identified clusters and their associated marker genes were classified into eight discrete cell groups. Finally, all cells along with the original count matrix for each cell population were extracted, enabling the reconstruction of the Seurat project and the subsequent annotation of cellular subpopulations.

To delineate subclusters within each principal cell type, we conducted a secondary clustering analysis on T cells, B cells, NK cells, DCs, and monocytes individually. This secondary clustering procedure mirrored the initial clustering approach, utilizing the previously selected HVGs as described above, with the resolution parameter varied between 0.01 and 1. The annotation of the resulting subclusters was performed by referencing established marker genes and the top differentially expressed genes relative to other cell populations (Milosevic et al. 2022).

### 2.5 Identification of Phenotype‐Associated Cell Cluster

The Single‐Cell Identification of Subpopulations with Bulk Sample Phenotype Correlation (Scissor) method was developed to pinpoint cell subsets that exhibit the strongest association with a specific phenotype by integrating single‐cell and bulk RNA sequencing data (Sun et al. 2022). In brief, by utilizing a bulk RNA expression matrix coupled with phenotypic data from centenarians alongside a single‐cell RNA sequencing dataset, we successfully identified cell subpopulations that are most significantly correlated with previously uncharacterized features within the single‐cell dataset. The algorithm classifies cells as Scissor positive (Scissor^+^) or Scissor negative (Scissor^−^), corresponding to positive and negative associations with the phenotype of interest, respectively. By applying this approach to a single‐cell expression matrix related to aging and the bulk expression matrix containing centenarian phenotypic information, we were able to delineate aging‐associated cell subsets that are most strongly linked to longevity.

### 2.6 Pseudotime Analysis

To further elucidate the mechanisms underlying healthy longevity in centenarians, trajectory analysis was conducted utilizing Monocle 3. Initially, the cell population with the highest proportion of Scissor‐positive and Scissor‐negative cells was identified and selected for Monocle analysis. Subsequently, principal component analysis (PCA) was performed using HVGs, and the top 50 genes exhibiting the strongest positive and negative correlations with the top 10 principal components, based on gene loadings, were employed to order the cells along a pseudotemporal trajectory. Dimensionality reduction and visualization were then carried out using the “DDRTree” and “plot_cell_trajectory” functions within the Monocle 3 package. Additionally, Monocle 3 was utilized to depict the cellular differentiation process.

### 2.7 Differentially Expressed Genes (DEGs) Identification and Functional Enrichment Analysis

Differential expression analysis was conducted between Scissor‐positive (Scissor^+^) and Scissor‐negative (Scissor^−^) cells identified within a single‐cell transcriptomic dataset, utilizing the “FindMarkers” function in Seurat following the exclusion of megakaryocyte and erythrocyte populations. The gene with absolute log_2_FC threshold > 0.5 and *p* value < 0.05 was considered as a hub gene. Volcano plots illustrating the differential expression of these genes between groups were generated using the ggplot2 package in R. Subsequently, Gene Ontology (GO) and Kyoto Encyclopedia of Genes and Genomes (KEGG) pathway enrichment analyses of the identified hub genes were performed employing the ClusterProfiler package, in accordance with established methodologies, to elucidate their potential involvement in longevity status (Yu et al. 2012). The enrichment results were visualized as bar plots using ggplot2.

### 2.8 Genes of Centenarians Screening and Co‐Localization Analysis

Aging‐associated genes were obtained from the CellAGE database (https://genomics.senescence.info/cells/). Following the removal of duplicate entries, a total of 866 unique genes associated with senescence were identified. DEGs overlapping with these genes associated with senescence were excluded from further analysis. The remaining DEGs were subsequently subjected to co‐localization analysis. The genome‐wide association study (GWAS) summary data comprised cohorts involved in previously published GWAS on longevity, including 3484 individuals aged over 99 years and 25,483 controls who died before reaching 60 years of age (Ni et al. 2023). Comprehensive cis‐expression quantitative trait loci (cis‐eQTL) summary statistics were retrieved from the single‐cell eQTLGen Consortium database (https://eqtlgen.org/sc/). Single nucleotide polymorphism (SNP) loci were extracted within a 500‐kilobase window upstream and downstream of each gene. The prepared datasets from eQTL and GWAS were then utilized for co‐localization analysis using the R package “coloc.” This approach employed a Bayesian framework to evaluate five hypotheses (Wang et al. 2020): H0, indicating no association between eQTL and GWAS traits in the region; H1, where only eQTL is associated; H2, where only GWAS is associated; H3, where both are associated but influenced by distinct causal SNPs; and H4, where both traits are associated and share the same causal SNP, implying co‐localization. GWAS loci overlapping with eQTL sites were selected for co‐localization testing. The posterior probability of hypothesis 4 (PP.H4), representing the likelihood that both traits share the same causal variant, was calculated within ±500 kb of the lead GWAS SNP. Events with PP.H4 values exceeding 0.7 were considered indicative of significant GWAS‐eQTL co‐localization.

## 3 Results

### 3.1 Single‐Cell Transcriptome Landscape in Aging Cohort

**FIGURE 1:**
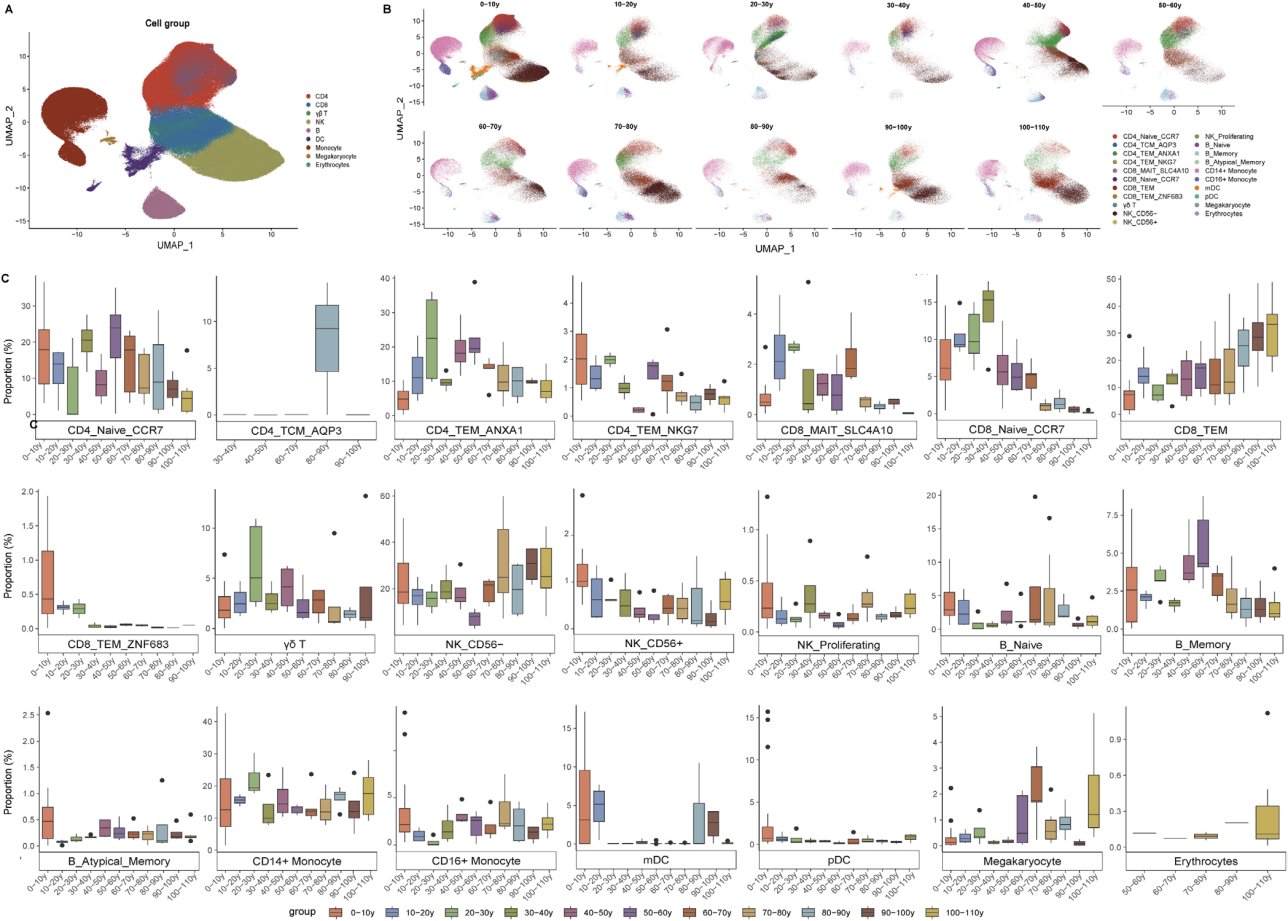
Single‐cell transcriptome landscape in aging cohort. (A) UMAP visualization of cell‐type‐specific annotation among the aging cohort, showing 9 cell groups in different colors. (B) UMAP visualization of immune cell subpopulation annotation across different age groups, displaying 21 subpopulations in different colors. (C) The proportion of 21 different cell types across age groups.

We obtained two single‐cell RNA sequencing (scRNA‐seq) datasets generated by 10× Genomics, comprising samples from 68 healthy individuals aged between 0 and 110 years from East Asia (Table S1). Data preprocessing was conducted using the Seurat R package. After 5402 cells were excluded through quality control, a total of 559,713 cells were identified (Figure S1A). Then we accounted for technical differences and integrated multiple samples using R package Harmony. During the process of scaling the data, we identified the top 2000 HVGs after all ribosomal and mitochondrial genes were removed (Figure S1B). Subsequently, the samples were categorized into 11 distinct groups, each corresponding to a decade of age (Figure S1C). Following this, in the dimensionality reduction phase of integrated data, the elbow‐shaped plot indicates that the ideal number of principal components (PCs) is 10 (Figure S1D). Louvain graph‐based clustering with a resolution parameter set to 0.1 was performed to group cells into populations of similar expression profiles. Nine distinct cell clusters were identified, including CD4^+^ T cell, CD8^+^ T cell, γδ T cell, NK cell, B cell, DC, Monocyte, Megakaryocyte, and Erythrocytes, and visualized by Uniform Manifold Approximation and Projection (UMAP) (Figure 1A,B). Markers for defining these cell clusters were shown in Figures S2 and S3. Further annotation of cell clusters into subpopulations was achieved by varying principal components and resolution parameters, resulting in the identification of 21 distinct cell subgroups (Figure 1C). The expression patterns of 42 marker genes across different age groups are illustrated in Figure S4.

In summary, the relative abundances of the CD8_TEM and NK_CD56^−^ cell populations were significantly higher in the 90–100 and 100–110 year age cohorts compared to other age groups. In contrast, the frequencies of the CD8_MAIT_SLC4A10 and CD8_Naive_CCR7 cell populations were notably reduced in these same age groups relative to those observed in younger cohorts. Additionally, the proportions of CD4_Naive_CCR7, B_Naive, and B_Memory cells were substantially lower in the 90–100 and 100–110 year age groups compared to other age categories.

### 3.2 Identification of Phenotype‐Associated Cell Type and Pseudotime Analysis to Illustrate the Process of Cell Differentiation

**FIGURE 2:**
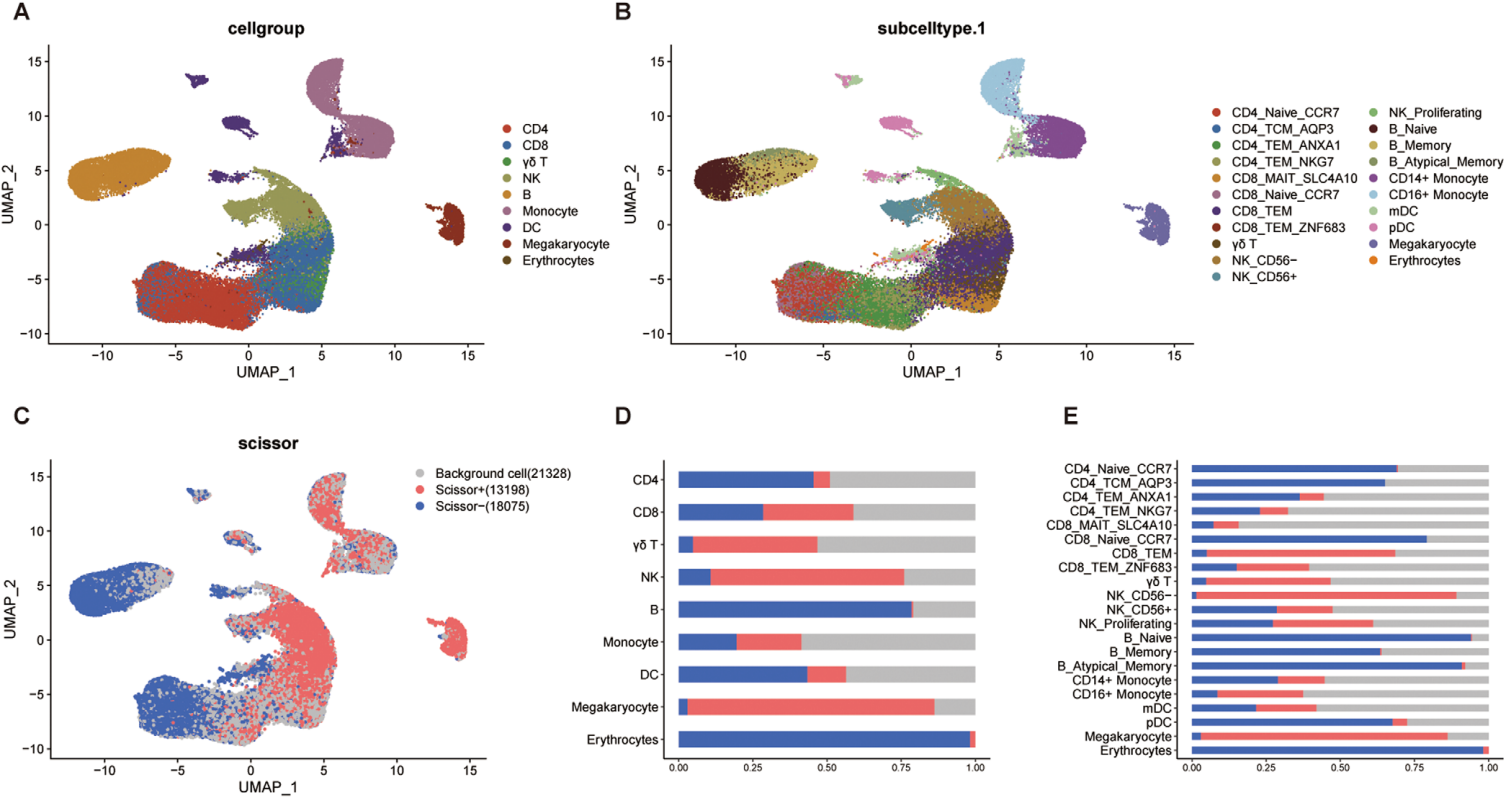
Centenarian phenotype‐associated immune cell type analysis at single‐cell resolution. (A) UMAP visualization of cell‐type‐specific annotation among immune cells, showing 9 cell groups in different colors. (B) UMAP visualization of subcellular annotation among immune cell subpopulations, showing 21 subpopulations in different colors. (C) UMAP visualization of Scissor^+^ and Scissor^−^ cells. (D, E) Proportional fractions of identified cell types across Scissor^+/−^ conditions among extracted immune cells.

To advance the understanding of cellular contributions to centenarian development, we integrated single‐cell sequencing data and bulk RNA sequencing employing the R package Scissor. Given the large number of cells, a subset of 5000 cells was randomly selected from each distinct cell cluster. The analysis incorporated a total of 52,601 cells, and the dataset was subsequently reconstructed to enable standardization, homogenization, and dimensionality reduction using UMAP and t‐SNE methodologies (Figure 2A,B). This approach ultimately identified 13,198 Scissor^+^ cells, which exhibited a positive association with centenarians, 18,075 Scissor^−^ cells, and 21,328 background cells (Table S2). Notably, Scissor^+^ cells are predominantly accounted for in NK cells, CD8 T cells, γδ T cells, and megakaryocytes. Conversely, Scissor^−^ cells are primarily represented in CD4 T cells, B cells, DCs, and erythrocytes (Figure 2C–E).

**FIGURE 3:**
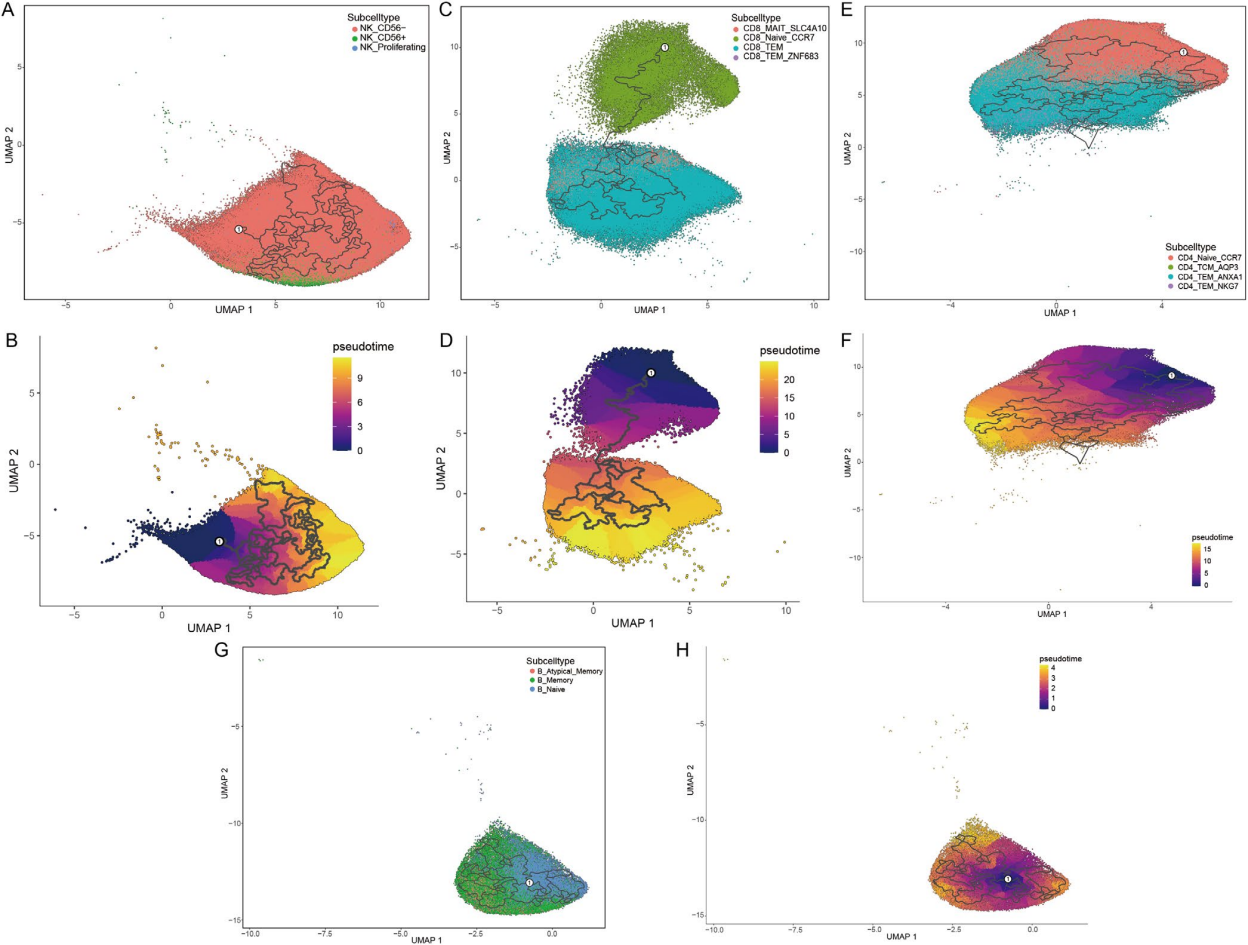
Clustering and pseudotime analysis of four cell types. (A, B) UMAP visualization of NK cell subpopulations and pseudotime trajectory. (C, D) UMAP visualization of CD8^+^ T cell subpopulations and pseudotime trajectory. (E, F) UMAP visualization of CD4^+^ T cell subpopulations and pseudotime trajectory. (G, H) UMAP visualization of B cell subpopulations and pseudotime trajectory.

By integrating the distribution of cell populations within the age ranges of 90–100 and 100–110 years, alongside the results obtained from Scissor analysis, we conducted pseudotime analyses on the NK and CD8 T cell clusters, respectively. Our findings indicate that the differentiation trajectories of NK cells can be classified into three distinct subsets: NK_CD56^−^, NK_CD56^+^, and NK_Proliferating (Figure 3A,B). Conversely, the differentiation pathways of CD8 T cells are characterized by two separate trajectories. The first trajectory progresses from CD8_Naive_CCR7 and CD8_TEM_ZNF683 populations toward CD8_TEM cells, whereas the second trajectory extends from CD8_Naive_CCR7 to CD8_MAIT_SLC4A10 cells (Figure 3C,D). The differentiation timeline for CD4 T cells follows a sequential progression from CD4_Naive_CCR7 to CD4_TCM_AQP3, then to CD4_TEM_ANXA1, and finally to CD4_TEM_NKG7 (Figure 3E,F). Similarly, the differentiation trajectory of B cells proceeds from B_Naive to B_Memory and subsequently to B_Atypical_Memory subsets (Figure 3G,H).

### 3.3 Functional Enrichment Analysis of Hub Genes

**FIGURE 4:**
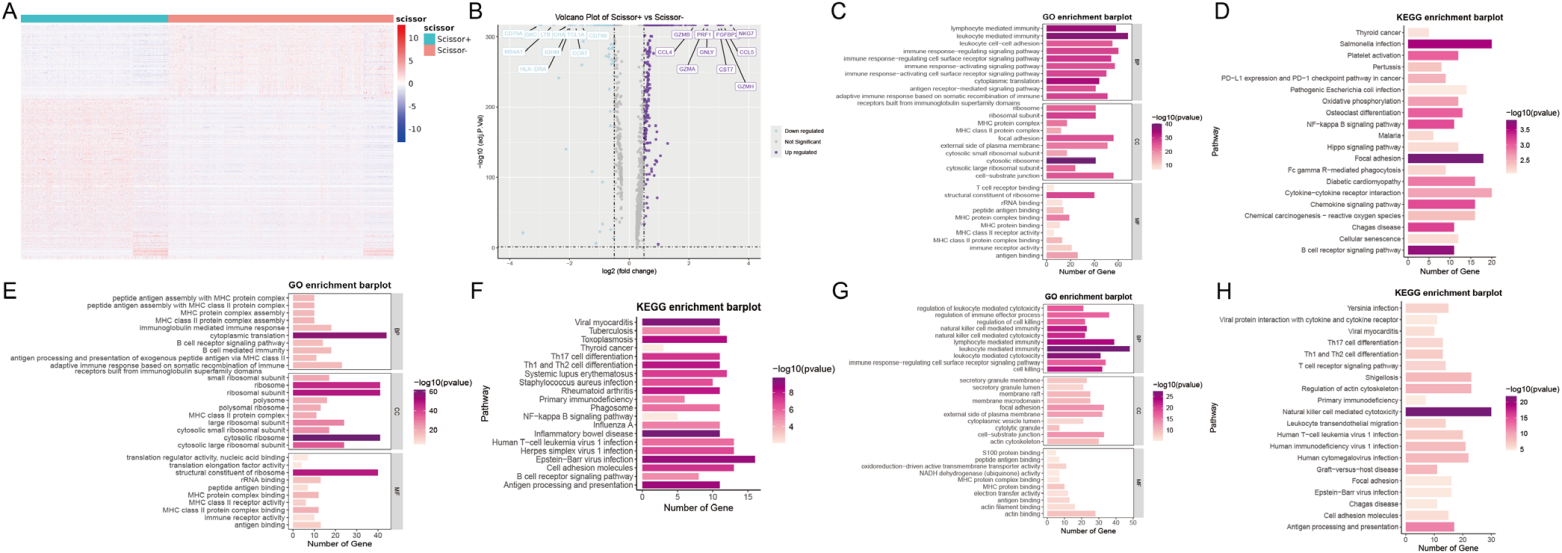
Differentially expressed genes and functional enrichment. (A) Heatmap of differential gene expression between Scissor^+^ and Scissor^−^. (B) Volcano plot of differential gene expression between Scissor^+^ and Scissor^−^. (C, D) GO and KEGG enrichment analysis of all differentially expressed genes. (E, F) GO and KEGG enrichment analysis of differentially expressed genes in Scissor^−^ cell cluster. (G, H) GO and KEGG enrichment analysis of differentially expressed genes in Scissor^+^ cell cluster.

To elucidate the transcriptional profiles underlying the cell populations associated with centenarians, we conducted a comparative analysis of gene expression between Scissor^+^ cell and Scissor^−^ cell clusters. In total, 317 significantly upregulated hub genes and 147 significantly downregulated hub genes were identified (Figure 4A, Table S3). The top 10 upregulated genes included GZMB, PRF1, FGFBP2, NKG7, CCL4, GZMA, GNLY, CST7, GZMH, and CCL5, whereas the top 10 downregulated genes comprised CD79A, GKC, LTB, JCHAIN, TCL1A, CD79B, MS4A1, HLA‐DRA, IGHM, and CCR7 (Figure 4B). Subsequent gene enrichment analyses were performed on these hub genes. Gene Ontology Biological Process (GO‐BP) enrichment revealed significant involvement in lymphocyte‐mediated immunity (GO:0002449), leukocyte‐mediated immunity (GO:0002443), and cytosolic ribosome components (GO:0022626) (Figure 4C, Table S4). The KEGG pathway analysis indicated enrichment in the NF‐κB signaling pathway (hsa04064), Hippo signaling pathway (hsa04390), cytokine‐cytokine receptor interaction (hsa04060), chemokine signaling pathway (hsa04062), and B cell receptor signaling pathway (hsa04662) (Figure 4D, Table S5). Further GO‐BP enrichment analysis of the Scissor^−^ cell cluster highlighted its predominant involvement in inflammatory processes, including cytoplasmic translation (GO:0002181) and structural constituents of the ribosome (GO:0003735) (Figure 4E, Table S6). KEGG pathway analysis corroborated these findings by demonstrating upregulation of inflammation‐related signaling pathways, such as Th17 cell differentiation (hsa04659), Th1 and Th2 cell differentiation (hsa04658), and NF‐κB signaling (hsa04064), consistent with the known molecular functions of CD4^+^ T cells (Figure 4F, Table S7). Conversely, the Scissor^+^ cell cluster was predominantly associated with regulation of NK cell‐mediated immunity (GO:0002715), NK cell‐mediated cytotoxicity (GO:0042267), lymphocyte‐mediated immunity (GO:0002449), and leukocyte‐mediated cytotoxicity (GO:0001909) (Figure 4G, Table S8). Pathway enrichment analysis further revealed significant enrichment of Th17 cell differentiation (hsa04659), T cell receptor signaling pathway (hsa04660), and NK cell‐mediated cytotoxicity (hsa04650) within the Scissor^+^ cell cluster (Figure 4H, Table S9). Collectively, the integrative analysis combining single‐cell and bulk RNA sequencing data via the Scissor algorithm identified a cell subpopulation most strongly correlated with immune remodeling. These findings underscore the pivotal role of the Scissor^+^ and Scissor^−^ cell clusters in mediating cytotoxic functions.

### 3.4 Screening and Colocalization Analysis of Genes Associated With Centenarians

**FIGURE 5:**
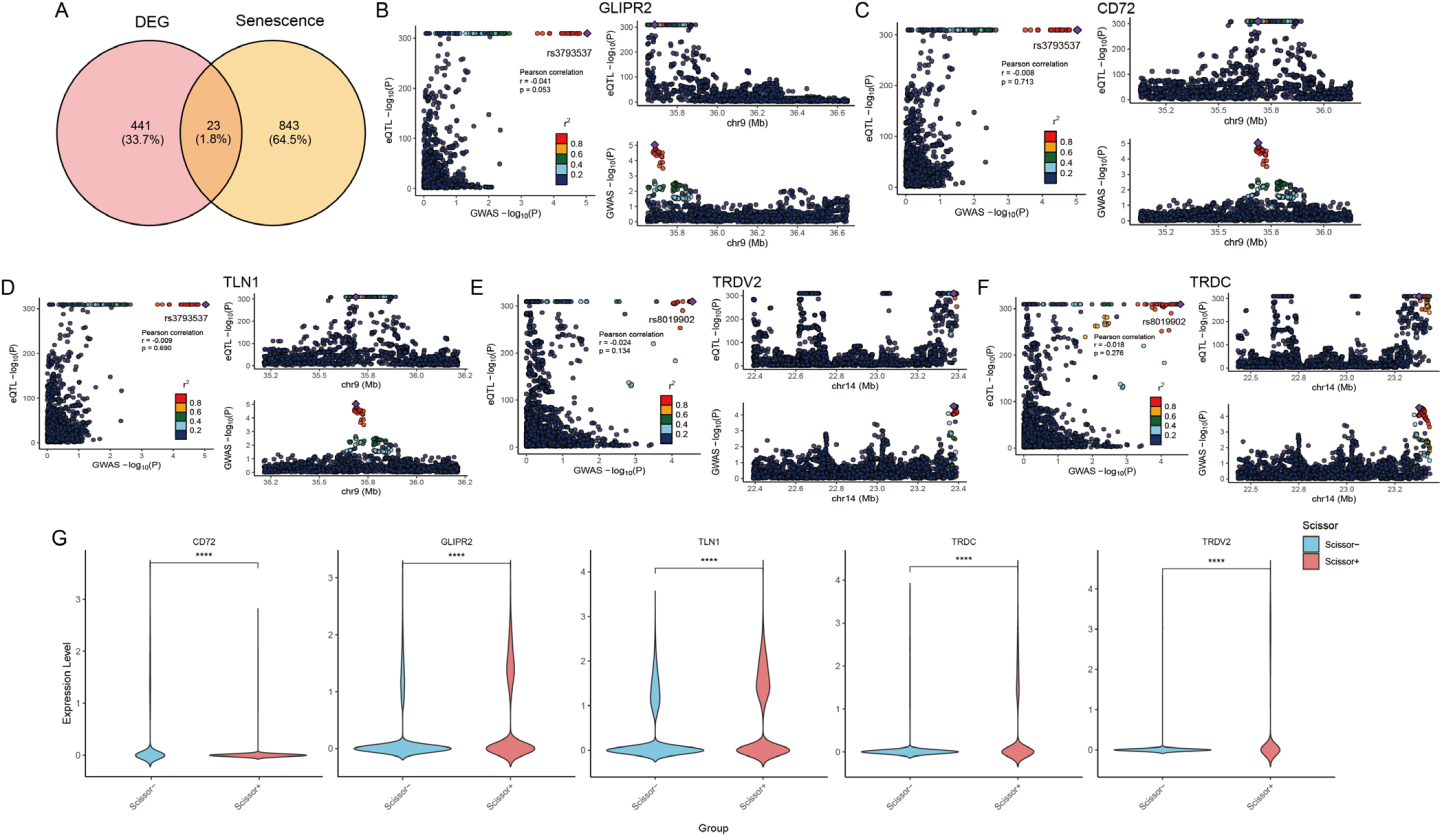
Genes of centenarians screening and co‐localization analysis. (A) Venn diagram of DEGs and aging‐related genes in Scissor^+^/Scissor^−^. (B–F) Five eQTL‐co‐localized events. (G) Expression of the five co‐localized genes in the Scissor^+^ and Scissor^−^ groups.

An intersection analysis was performed between 464 differentially expressed genes and those implicated in cell senescence, revealing that 441 of these genes were uniquely expressed in centenarians (Figure 5A). Subsequently, a single‐cell transcriptional expression analysis of these 441 genes was conducted, employing colocalization analysis based on the PP.H4 statistic across various centenarian phenotypes to assess whether the eGene‐trait associations were driven by a shared set of causal single nucleotide polymorphisms (SNPs) (Table S10). This analysis identified five eQTL‐colocalized events relevant to centenarians, each exhibiting a PP.H4 value greater than 0.7. Notably, rs3793537 emerged as the lead SNP colocalizing with the target genes GLIPR2, CD72, and TLN1 (Figure 5B–D), all located within an approximately 35 megabase region on chromosome 9. Furthermore, rs8019902 was identified as the lead SNP colocalizing with the target genes TRDV2 and TRDC (Figure 5E,F), both situated within an approximately 23 megabase region on chromosome 14. The expression patterns of these five colocalized genes in the Scissor^+^ and Scissor^−^ groups are presented in Figure 5G.

## 4 Discussion

This study conducted a comprehensive analysis of the fundamental features of immunosenescence in centenarians by integrating single‐cell and bulk transcriptomic datasets. Scissor^+^ cells were predominantly identified within NK cells, CD8 T cells, γδ T cells, and megakaryocytes. Functional enrichment analyses indicated that Scissor^+^ cells are primarily associated with immune regulatory pathways, underscoring the critical role of immune regulation in maintaining immune homeostasis. Pseudotime trajectory analysis further delineated the dynamic differentiation pathways of longevity‐associated cell subpopulations: NK cells progressed along a trajectory from CD56^−^ to CD56^+^ to proliferative states, whereas CD8 T cells followed two distinct differentiation routes, transitioning from naive cells to either effector memory or MAIT cells. In contrast, Scissor^−^ cells were mainly represented by CD4 T cells, B cells, DCs, and erythrocytes. Functional enrichment of Scissor^−^ cells revealed associations with inflammatory processes and T helper (Th) cell differentiation, highlighting a close relationship with CD4 T cells. Pseudotime analysis of longevity‐negative cell subpopulations demonstrated that CD4 T cells differentiate along a trajectory encompassing CD4_Naive_CCR7, CD4_TCM_AQP3, CD4_TEM_ANXA1, and CD4_TEM_NKG7 subsets, whereas B cells transition from naive to memory and subsequently to atypical memory subsets. Collectively, these pseudotime analyses indicate that the differentiation trajectories of longevity‐associated positive cell populations, including cytotoxic effector lineages such as NK cells, CD8^+^ cells and γδ T cells, exhibit functions consistent with pathway enrichment analyses, promoting the maintenance and renewal of immune surveillance as well as the elimination of damaged or senescent cells. Conversely, the differentiation pathways of longevity‐associated negative cell populations, comprising CD4^+^ T cells, B cells, and DCs, predominantly reflect states associated with helper T cell polarization and inflammatory responses, which is likewise corroborated by the pathway enrichment results. Differential gene expression analysis identified 464 phenotype‐associated genes, of which 441 were uniquely expressed in centenarians. Co‐localization analysis uncovered five colocalized events linked to longevity. Notably, rs3793537, the lead SNP identified in GWAS of centenarians, co‐localized with eQTLs for the genes GLIPR2, CD72, and TLN1. Additionally, rs8019902 was implicated through eQTL colocalization with the target genes TRDV2 and TRDC in the centenarian GWAS. Collectively, these results establish a conceptual framework aimed at preserving immune surveillance while mitigating maladaptive inflammaging processes during aging. Importantly, the observed cellular composition patterns align broadly with previous scRNA‐seq studies of long‐lived individuals, which similarly report an enrichment of cytotoxic effector populations alongside a relative reduction in helper/APC subsets. Beyond this corroboration, the current study contributes three notable advancements: (i) integrative analyses combining phenotypic data with single‐cell transcriptomics that more directly associate scRNA‐seq‐defined subpopulations with the longevity phenotype; (ii) pseudotime trajectory analyses providing dynamic evidence of differentiation pathways within these subpopulations; and (iii) eQTL‐GWAS colocalization analyses that prioritize candidate genetic variants (rs3793537 linked to TLN1, GLIPR2, and CD72, and rs8019902 associated with TRDV2/TRDC) for targeted mechanistic follow‐up.

Conventional case–control analyses of single‐cell transcriptomic data are frequently influenced by group labels and batch effects, which can introduce biases in the identification of immune cell subpopulations genuinely associated with specific phenotypes. Furthermore, these analyses generally focus on detecting differential expression between two groups without quantifying the extent to which particular subpopulations contribute to the overall phenotype, thereby complicating the elucidation of underlying molecular mechanisms. Consequently, there is a need for novel computational approaches that enhance the precision and reliability of identifying key immune cell subsets linked to longevity phenotypes. The Scissor algorithm offers an innovative framework for interrogating single‐cell data and has been successfully applied in diverse domains including oncology (Sun et al. 2022; Chen et al. 2023; Cheng et al. 2024), cardiovascular diseases (Sun et al. 2022), and neurodegenerative disorders (Sun et al. 2022). Scissor leverages bulk phenotype information as a reference to directly extract cells whose expression profiles co‐vary with the phenotype from single‐cell datasets, thereby obviating the need for manual grouping and mitigating batch effect confounders. In contrast, traditional case–control methodologies rely on initial grouping followed by comparative analyses, which are susceptible to inaccuracies arising from erroneous group labels or inadequate batch correction, potentially leading to misidentification of phenotype‐associated subpopulations. To our knowledge, this study represents one of the first applications of the Scissor framework to identify immune cell subpopulations associated with longevity‐related phenotypes in centenarians. By integrating the bulk transcriptomic “Longevity Molecular Tags” derived from the Guangxi centenarian cohort with single‐cell transcriptomic data comprising 52,601 PBMC single‐cell data, we identified 13,198 Scissor^+^ cells and 18,075 Scissor^−^ cells, directly pointing to the cell subpopulations positively and negatively associated with the longevity phenotype.

Scissor^+^ cells predominantly encompass NK cells, CD8^+^ T cells, and γδ T cells, all of which are classified within the “cytotoxic effector” lineage. In contrast, Scissor^−^ cells are chiefly composed of CD4^+^ T cells, B cells, and DCs. Together, these patterns suggest that exceptional longevity is associated with an adaptive immunosenescence program in which cytotoxic effector lineages are preserved and dynamically renewed to sustain immune surveillance and clearance of damaged/senescent cells, whereas helper and antigen‐presenting compartments are concomitantly tuned to restrain maladaptive inflammatory polarization. In this framework, centenarians may achieve “healthy aging” not by global immunosuppression, but by maintaining functional immune homeostasis through coordinated remodeling of the cytotoxic–helper axis, thereby limiting inflammaging while preserving protective immunity. Specifically, NK cells progress along a trajectory characterized by the transition from CD56^−^ to CD56^+^ to proliferative states, thereby continuously renewing their capacity to eliminate senescent or mutant cells while preserving their self‐expansion potential (Kaszubowska et al. 2018). Age‐associated alterations in NK cell subsets, notably the expansion of CD56^−^ and CD56^dim^ populations, have been implicated in adaptive remodeling processes underlying immunosenescence (Muller‐Durovic et al. 2019). CD8^+^ T cells differentiate via two distinct pathways: one leading to the generation of highly functional memory cells that enhance peripheral immune surveillance, and the other giving rise to MAIT cells that contribute to the maintenance of mucosal barrier homeostasis (Van Der Geest et al. 2018). γδ T cells exhibit dual roles encompassing pro‐inflammatory and immunoregulatory functions relevant to tumor immunity, tissue repair, and inflammation modulation. Their enrichment in centenarians suggests that the regulation of chronic inflammation may constitute a critical mechanism for promoting healthy longevity (Colonna‐Romano et al. 2004). Although γδ T cell functionality generally declines with age in the broader population, their sustained presence and altered activation states in centenarians may reflect compensatory adaptations that support immune regulation (Norenberg et al. 2020).

A comparative analysis between Scissor^+^ and Scissor^−^ cells identified 464 differentially expressed genes, of which 441 were uniquely associated with centenarians. Functional enrichment analysis indicated that the upregulated genes predominantly participate in inflammatory pathways, including NF‐κB, NOD‐like receptor, and TNF signaling. Conversely, the downregulated genes were primarily involved in extracellular matrix remodeling and TGF‐β signaling pathways. These findings suggest that centenarians preserve immune homeostasis by maintaining a state of “precise low‐grade inflammation” rather than through broad immune suppression.

The upregulation of TLN1, a critical protein implicated in cell adhesion and mechanotransduction, may augment the migratory capacity, tissue residency stability, and responsiveness of immune cells to antigenic and mechanical stimuli within the tissue microenvironment (Li et al. 2024). Such enhancement is essential for the effective surveillance, recognition, and elimination of damaged cells, including senescent and malignant cells, by immune cells in aging tissues. Additionally, the increased expression of TRDV2 and TRDC, integral components of the γδ T cell receptor—particularly the Vδ2 subset—indicates the expansion and/or functional activation of specific γδ T cell subpopulations in long‐lived individuals. This observation aligns with the established role of γδ T cells in innate immune surveillance, rapid stress response, clearance of senescent cells, and regulation of tissue inflammation (Poggi et al. 2007). The preservation of γδ T cell activity thus represents a fundamental protective mechanism against age‐related immune deterioration and chronic inflammatory conditions.

Previous research has predominantly characterized GLIPR2 as a negative regulator of autophagy, with its decreased expression being associated with the maintenance of basal autophagy levels and lifespan extension (Karagiannis et al. 2023; Parnes and Pan 2000). Contrarily, the present study observed an upregulation of GLIPR2 within specific immune cell populations linked to longevity, indicating that its functional role may be highly dependent on cell type or anatomical context. In immune cells, particularly those exhibiting potent cytotoxic or surveillance activities such as NK cells, CD8^+^ T cells, and γδ T cells, the increased expression of GLIPR2 may extend beyond autophagy inhibition. It may contribute to the modulation of more intricate processes including immune cell activation, survival, metabolic reprogramming, and membrane dynamics associated with effector functions. Consequently, GLIPR2 likely plays a critical role in preserving the functional stability and adaptability of immune cells, thereby facilitating sustained immune surveillance and stress response mechanisms that underpin healthy aging and longevity.

By integrating multi‐scale findings derived from Scissor analysis, this study presents a comprehensive characterization of the compensatory adaptations within the immune system of centenarians at the cellular level (notably NK, CD8, and γδ T cell predominance), the transcriptional level (highlighting a precise inflammatory regulatory module), and the genetic level (identifying SNPs at loci 9q34.2 and 14q11.2). These results provide novel insights for the development of therapeutic strategies aimed at modulating NK‐T cell functionality or enhancing γδ T cell receptor diversity to mitigate aging‐related decline. Despite its contributions, this study is constrained by its cross‐sectional design, relatively limited sample size, and the absence of validation across ethnically diverse populations. Moreover, cytomegalovirus (CMV) serostatus may confound the interpretation of age‐associated immune alterations. Future investigations will incorporate CMV IgG serological testing and employ methodologies such as CMV‐stratified analyses or adjustment for CMV serostatus as a covariate to more precisely disentangle immune remodeling attributable to CMV from immune signatures related to longevity. Additionally, although our integrative analyses prioritized SNP‐linked regulatory signals and candidate genes and pathways, the putative regulatory mechanisms (e.g., cis‐regulatory effects) and downstream functional results remain to be empirically validated. The potential translation of these molecular signatures into clinically actionable biomarkers also warrants further assessment. Consequently, subsequent research should integrate external replication in larger, multi‐ethnic cohorts with longitudinal multi‐omics profiling and comprehensive functional validation at multiple levels (including perturbation experiments, protein‐level verification, and in vitro and in vivo models) to elucidate temporal dynamics, enhance causal inference, and clarify the mechanistic roles of gene–immune regulatory networks in healthy aging. Specifically, priority will be given to validating key candidate genes and functions identified herein, such as TLN1‐associated adhesion/mechanotransduction programs, TRDV2/TRDC‐related γδ T‐cell programs, and the context‐dependent regulation of GLIPR2.

## 5 Conclusion

This study develops an immune cell atlas of “Longevity Molecular Tag” through the integration of single‐cell and bulk transcriptomic data. The findings indicate that centenarians attain an immune equilibrium conducive to healthy longevity via the remodeling of cytotoxic immune cell lineages and the fine‐tuning of inflammatory responses, rather than through generalized immunosuppression. Our integrative Scissor framework, which couples lifespan single‐cell transcriptomes with bulk transcriptomic profiles from Guangxi centenarians, identifies longevity‐associated Scissor^+^ subpopulations predominantly within NK cells, CD8^+^ T cells, γδ T cells, and megakaryocytes, and longevity‐negative Scissor^−^ subpopulations mainly within CD4^+^ T cells, B cells, DCs, and erythrocytes; it further prioritizes genetically supported candidate axes based on eQTL–GWAS colocalization (rs3793537–GLIPR2/CD72/TLN1 and rs8019902–TRDV2/TRDC) as putative molecular targets linked to extended lifespan, thereby offering novel avenues for interventions aimed at fostering healthy aging.

## Author Contributions

Z.Z., H.S., and S.F. statistical analysis, visualization, and interpretation of data, writing – original draft, and critical revision. F.J., W.S., L.L. and E.L. data collection, methodology, statistical analysis, visualization, and interpretation of data. C.H., L.W., and Y.L. data collection. X.N. conceptualization, supervision, project administration. All authors contributed to subsequent versions of the manuscript and approved the final version of the manuscript.

## Funding

The authors have nothing to report.

## Conflicts of Interest

The authors declare no conflicts of interest.

## Supporting information


**Figure S1:** Quality control and integration normalization results of two single‐cell datasets.

**Figure S2:** UMAP visualization of 42 marker genes.

**Figure S3:** Expression of 42 marker genes across 21 cell types.

**Figure S4:** Expression profile of 42 marker genes across different age groups.

**Table S1:** Baseline characteristics of data samples derived from single‐cell transcriptome sequencing.
**Table S2:** Summary of Scissor^+^ and Scissor^−^ cell proportion across immune cell subpopulations.
**Table S3:** Differentially expressed genes between Scissor^+^ and Scissor^−^ cells.
**Table S4:** GO‐BP enrichment analysis of differentially expressed genes.
**Table S5:** KEGG enrichment analysis of differentially expressed genes.
**Table S6:** GO‐BP enrichment analysis of differentially expressed genes in Scissor^−^ cell cluster.
**Table S7:** KEGG enrichment analysis of differentially expressed genes in Scissor^−^ cell cluster.
**Table S8:** GO‐BP enrichment analysis of differentially expressed genes in Scissor^+^ cell cluster.
**Table S9:** KEGG enrichment analysis of differentially expressed genes in Scissor^+^ cell cluster.
**Table S10:** eQTL‐Colocalized events associated with longevity phenotype.

## Data Availability

Raw single‐cell RNA sequencing data (56 healthy participants aged 0 to ≥ 90 years) generated in this study was deposited at Synapse repository (syn61609846). These data were derived from the following resources available in the public domain: doi: http://doi.org/10.1038/s41590‐024‐02059‐6. Raw UMI counts and normalized expression values for single cell RNA sequencing (7 supercentenarians and 5 controls) are publicly available at http://gerg.gsc.riken.jp/SC2018/. These data were derived from the following resources available in the public domain: doi: http://doi.org/10.1073/pnas.1907883116. The aging‐related genes were downloaded from the CellAGE network (https://genomics.senescence.info/cells/). Full cis‐eQTL summary statistics can be downloaded from the single‐cell eQTLGen Consortium database (https://eqtlgen.org/sc/).
