# Supplementary figures and images for: The Immune Cell Atlas of “Longevity Molecular Tag”: Identification of Principal Immune Cell Subsets and Their Underlying Molecular Regulatory Mechanisms
Source: Aging Cell. 2026 Mar 5;25(3):e70431. doi: 10.1111/acel.70431 (PMC12961527; doi:10.1111/acel.70431)

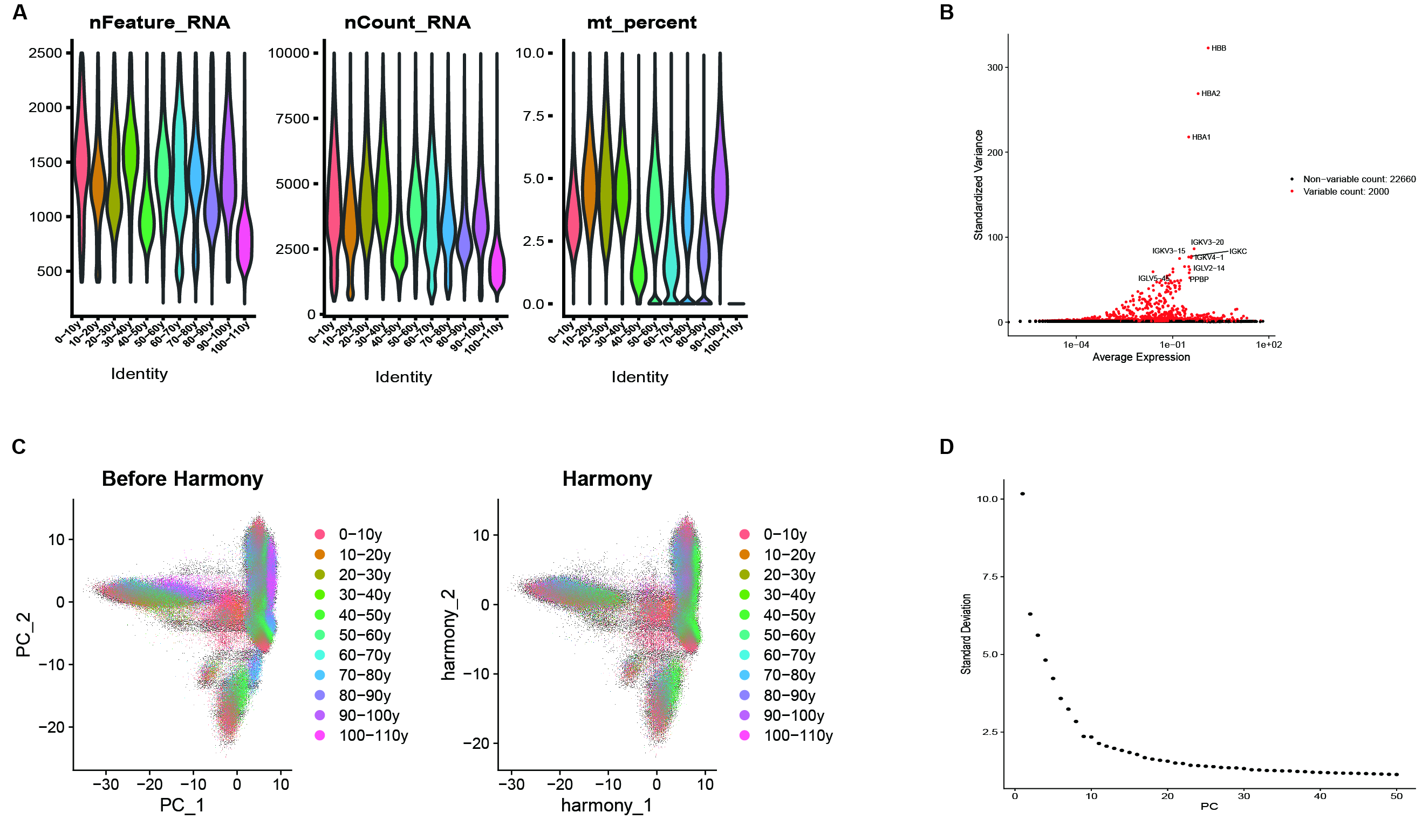

Supplement: Supplementary file 1 — Figure S1: Quality control and integration normalization results of two single‐cell datasets. [file ACEL-25-e70431-s004.tif]

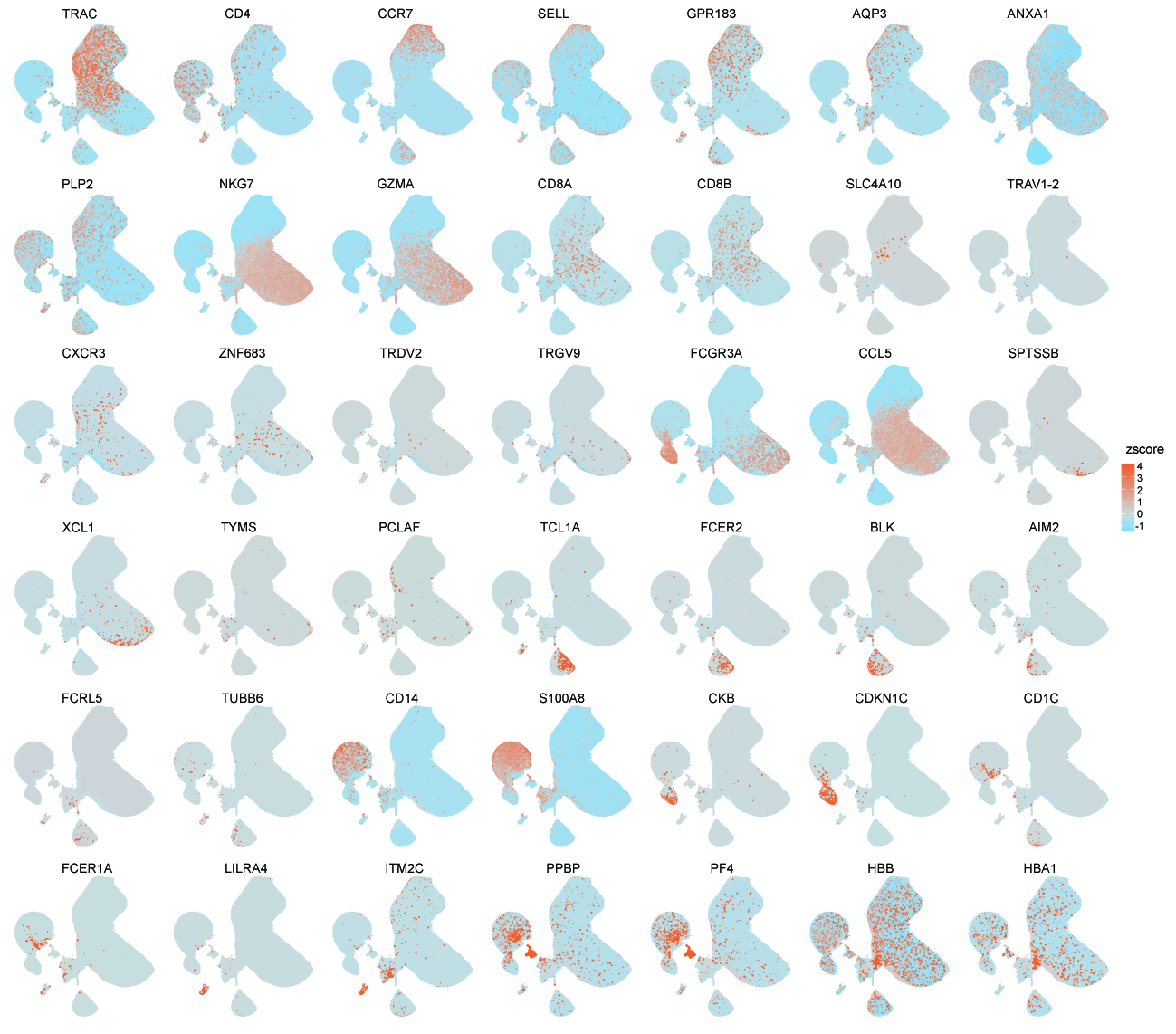

Supplement: Supplementary file 2 — Figure S2: UMAP visualization of 42 marker genes. [file ACEL-25-e70431-s001.tif]

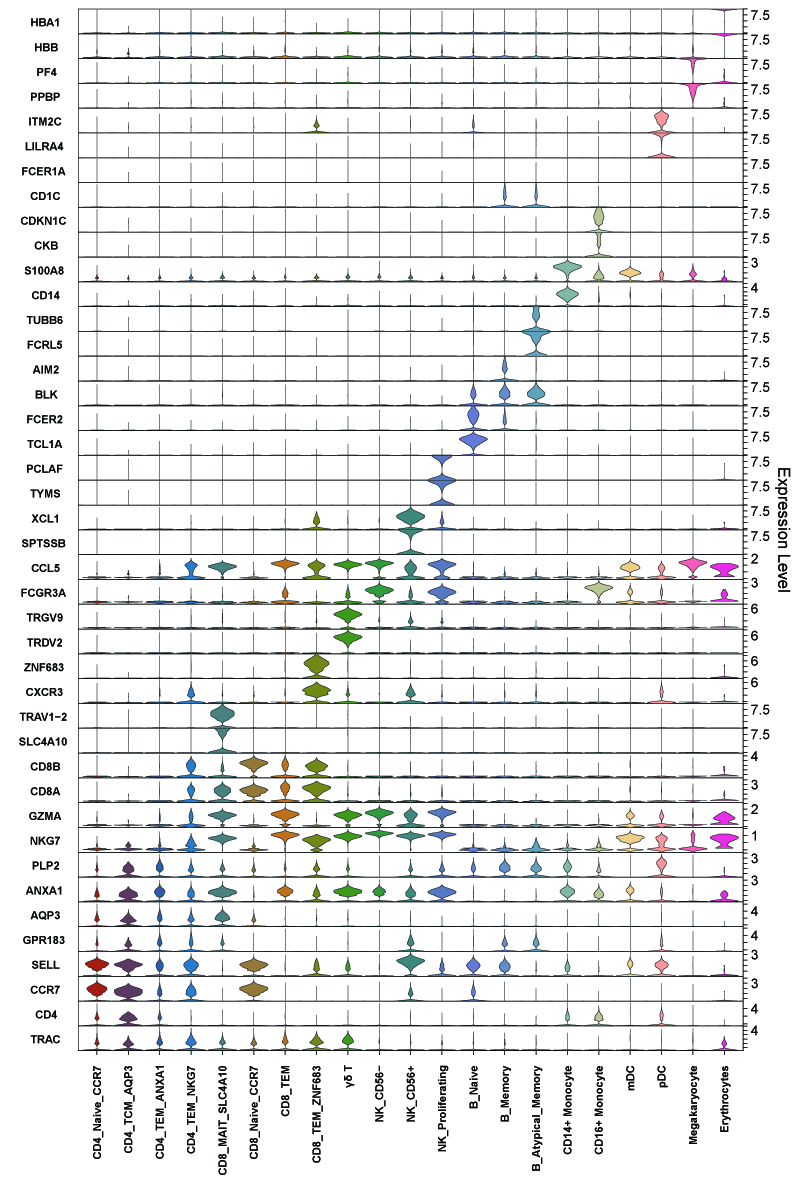

Supplement: Supplementary file 3 — Figure S3: Expression of 42 marker genes across 21 cell types. [file ACEL-25-e70431-s002.tif]

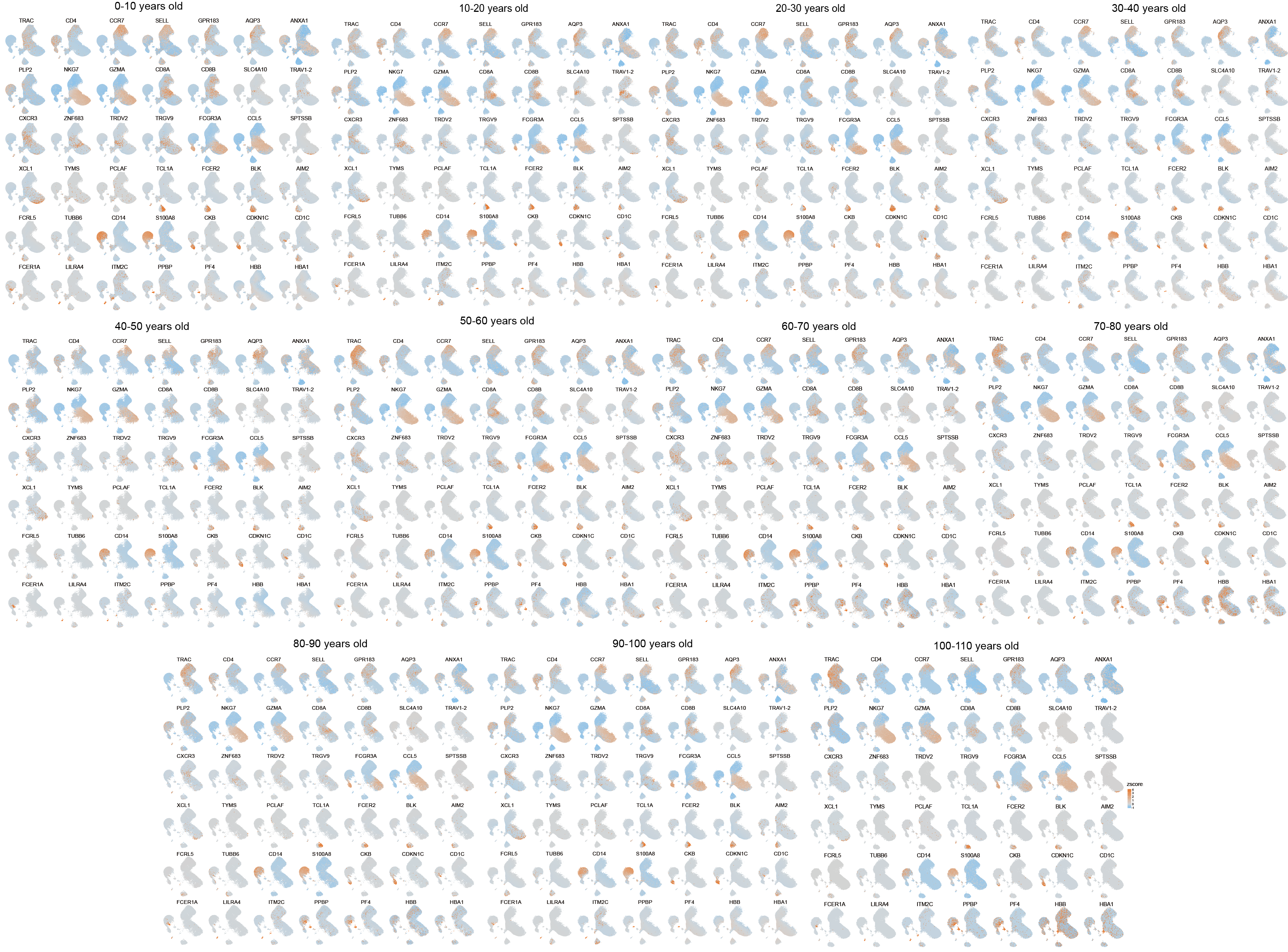

Supplement: Supplementary file 4 — Figure S4: Expression profile of 42 marker genes across different age groups. [file ACEL-25-e70431-s003.zip › acel70431-sup-0004-FigureS4.png]

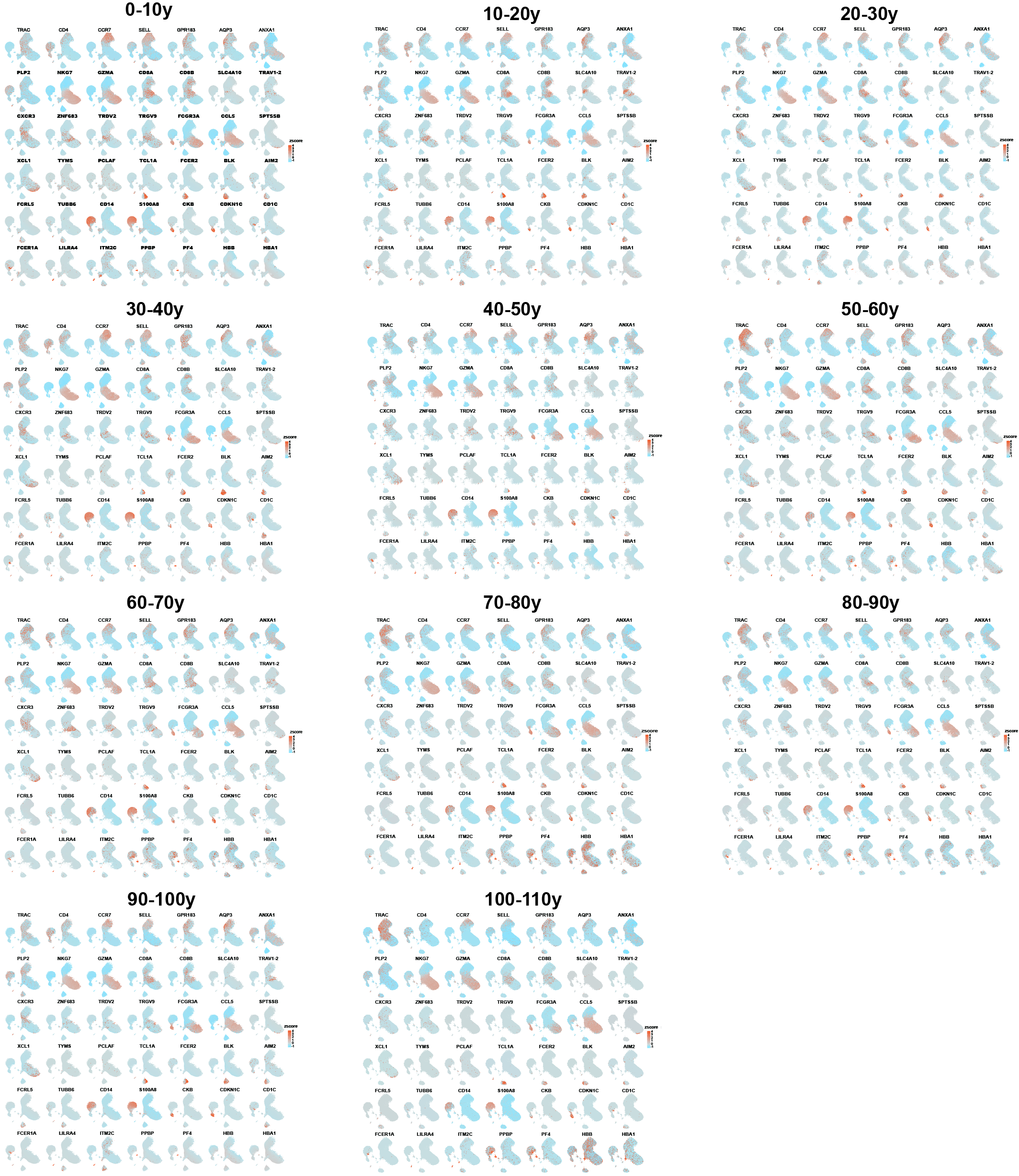

Supplement: Supplementary file 4 — Figure S4: Expression profile of 42 marker genes across different age groups. [file ACEL-25-e70431-s003.zip › acel70431-sup-0006-FigureS4@Figure S4.tif]
